# 

**Published:** 1897-01-23

**Authors:** 


### EARLY EDITION.

**Notices of Births, Deaths, and Marriages** are inserted in this column at a charge of 2s. 6d. for an announcement not exceeding four lines.

### NOTICE TO CORRESPONDENTS.

All MS., Letters, Books for Review, and other matters intended for the Editor should be addressed THE EDITOR, "THE HOSPITAL," THE HOSPITAL BUILDING, 28 & 29, SOUTHAMPTON STREET, STRAND, W.C.

The Editor cannot undertake to return rejected MS., even when accompanied by stamped directed envelo e.

**Notice to Advertisers and Subscribers.**

All Advertisements, Orders for Copies of THE HOSPITAL, and Business Communications should be addressed to **THE MANAGER, (Not to The Editor),** THE HOSPITAL, The Hospital Building, 28 & 29, Southampton Street, Strand, London, W.C.

The Cost of Subscription, post free, is as follows:—Per week, 2½d.; per quarter, 2s. 8d.; per half-year, 5s. 3d.; per annum, 10s. 6d. Foreign:—Per annum, 15s. 2d.; payable, in all cases, in advance.

**NOTICE.—Vol. XX. of "The Hospital" (April to September, 1896), in handsome cloth binding, is now on sale at the Office of this Journal, price 7s. 6d. Cases for binding the Numbers contained in this Volume, 1s. 6d. Index to Vol. XX., 2½d., post free.**

**The Monthly Part for February will be ready on February 1st. Price 6d., by post 8d.**

### SCRAPS AND GLEANINGS.

A BUILDING suitable for a convalescent home with accommodation for from 25 to 30 patients is to be leased by the Leeds Workpeople's Hospital Fund.

SIR HENRY E. ROSCOE, F.R.S., D.C.L., has accepted the post of English adviser of the Scientific Department of Liebig's Extract of Meat Company (Limited).

A NEW branch of the Nottingham General Dispensary has been opened at 130, Gregory Boulevard for the use of the inhabitants of the Hyson Green and Sherwood Rise Districts.

THE new district fever hospital at Falkirk, erected by the Eastern District Committee of the Stirling County Council, has been opened. The total cost of the hospital, including furnishings, was £7,500.

As the result of charge of 1d. admission (adults only) to their Christmas bazaar, the firm of J. R. Roberts Stores (Limited) have been enabled to hand over to the authorities of the West Ham Hospital the sum of £102 10s. 3d.

THE Budleigh Salterton Cottage Hospital, which was established as a Jubilee memorial, is appealing for further funds in order to make it a "model cottage hospital" as a memorial to the sixtieth year of the Queen's reign.

DURING the year 1895-6 the total number of patients in attendance at the Belfast Eye, Ear, and Throat Hospital numbered 1,493, of which 235 were in-patients. The receipts from all sources were £610, and the disbursements £609.

THE following figures showing the details of the Norwich Hospital Sunday and Saturday Fund Collections for the past three years are interesting; Sunday collections, 1894, £553; 1895, £639; 1896, £640. Saturday collections, 1894, £403; 1895, £442; 1896, £456. Street collections, 1894, £194; 1895, £169; 1896, £161. Total amount for division, 1894, £1,118; 1895, £1,225; 1896, £1,233. The school collections amounted to about £[illegible]2 each year.

THE total cost of maintenance of the Royal Albert Hospital, Devonport, for the year 18[illegible]5-6, has been £3,820.

THE result of the five days' bazaar in aid of the Kettering and District General Hospital was most satisfactory, a cheque for £3,000 being handed over to the Treasurer as the net proceeds.

THE total income of the Kent and Canterbury Hospital for the year 1896 was £3,847, and the expenditure £3,527. Six hundred and forty-two in-patients, 807 out-patients, and 1,073 casualties were admitted to treatment.

MR. STUDT, who for the past three years has organised a fête in aid of the Swansea Hospital, thinks that the committee of that hospital should take the financial responsibility of the fête which he was asked to hold this year on their own shoulders. This they have declined to do, but a town's meeting is to be called to consider the matter.

SINCE the first day of this year the official connexion heretofore existing between the Sunderland Provident Dispensary and the Sunderland Infirmary has been severed, the committee of the latter considering that it was desirable that the treatment of such cases as had hitherto been sent to the Provident Dispensary should be under the direct control of the Infirmary staff.

THE last report of the Erith House Institution for Invalid Ladies, Torquay, is not a satisfactory one from a financial point of view. "The cause of the decline in the pecuniary resources of Erith House," say the committee, "is palpable. The number of ladies visiting the House has of late years steadily decreased, until during the past season only twenty ladies availed themselves of its privileges, as against 30, 31, and 33 respectively in the three previous years, which probably may be accounted for by the mildness of last winter and also by the opening of similar institutions in other health resorts." Further on in the report the committee fear that unless a change for the better takes place "the question of the continued maintenance of Erith House will call for serious consideration."

### THE STUDENT OF MEDICINE.

### APPOINTMENTS.

R. Boyd, M.B., C.M.Glasg., appointed Senior House Surgeon to the Huddersfield Infirmary. H. D. Brook, L.R.C.P.Lond., M.R.C.S.Edin., appointed Medical Officer for the Second District and of the Workhouse of the Fareham Union. R. Burgess, M.B., C.M.Aberd., appointed Third House Surgeon to Huddersfield Infirmary. Dr. J. Easton, appointed Medical Officer of Health to the Weardale Rural District Council. J. G. Emanuel, M.R.C.S., L.R.C.P.Lond., B.Sc.Lond., appointed House Physician to the City of London Hospital for Diseases of the Chest, Victoria Park, E. J. P. Henry, B.A., M.D., P.Ch.Dub., L.M.R.C.P.I., appointed Divisional Surgeon to the Lewisham Police. Dr. T. H. Hughes, appointed Medical Officer of Health to the Connah's Quay Urban Council. M. B. James, M.B., Ch.B.Vict., appointed Second House Surgeon to Huddersfield Infirmary. J. H. Marsh, M.R.C.S., L.R.C.P., appointed Medical Officer and Ambulance Lecturer to the Macclesfield School Board. R. P. Shearer, M.B., C.M.Glasg., reappointed Medical Officer for the Gotham District of the Basford Union. E. W. Short, L.R.C.P., L.R.C.S.Edin., appointed Medical Officer for the No. 2 District of the Alston with Garrigill Parish. G. F. Smith, M.R.C.S., L.R.C.P., appointed Honorary Medical Officer to the Watford District Cottage Hospital. Dr. Stephenson, appointed Assistant Medical Officer to the Glasgow Parish Council. F. J. Walker, M.D., appointed Medical Officer of the Workhouse and Spilsby West District and Public Vaccinator, Spilsby Union. G. Watkins, M.A., M.B., B.C.Cantab., appointed Public Vaccinator for the City of Lincoln. G. F. West, L.R.C.P., L.R.C.S.Edin., appointed Resident Medical Superintendent to the Kilkenny District Asylum. H. J. M. Wyllys, L.R.C.P., L.R.C.S.Edin., appointed Junior House Surgeon to the Norfolk and Norwich Hospital.

### NEW AND STANDARD TEXT-BOOKS.

Crown 8vo., Cloth, with nearly 100 Illustrations. Price 2s. 6d.

**ELEMENTARY ANATOMY AND SURGERY FOR NURSES.** By William McAdam Eccles, M.S.Lond., M.B., F.R.C.S., L.R.C.P., Surgeon, West London Hospital; late Senior Assistant Demonstrator of Anatomy, St. Bartholomew's Hospital, &c., &c.

"Concise and eminently practical little book. . . . Nurses will find here just the information they need in the plainest possible language"—*Medical Magazine.*

"What is taught is simple in its language and style, and useful and practical in the matter submitted for the nurse's instruction. It is very freely illustrated . . . and the illustrations are exceedingly simple, well-defined, and intelligible."—*Glasgow Herald.*

"The book is well illustrated, and will be valuable to nurses who are beginning the study of anatomy. The instruction is given very clearly and concisely, and the reader is able to glean much information in a very condensed form."—*Nursing Notes.*

Crown 8vo., 500 pp., 2nd Edition, 7 Plates, 18 Illustrations, Charts, &c. 7s. 6d. net.

**NURSING.** By Isabel Adams Hampton. The Scientific Press have secured the English rights of this important work, and a stock of copies is on sale at their premises. It is considered one of the most important treatises on Nursing that has been published.

"It is not too much to say that in no single volume yet published has the subject been scientically, carefully, and completely treated as it has been by Miss Hampton."—*The Hospital.*

Demy 8vo., 200 pp., cloth gilt, profusely Illustrated with over 70 special Cuts. 6s.

**ART OF MASSAGE.** By A. Creighton Hale.

"Mrs. Hale seems to have had considerable experience not only as a practitioner, but as a teacher of the art, and explains in a series of clearly illustrated articles the many complaints for which massage has been found beneficial, and the special manipulations required in each case."—*Morning Post.*

"The latest and most complete book we know on Massage."—*Queen.*

Second Edition, Enlarged, Demy 8vo., Profusely Illustrated, nearly 200 pp., Cloth, 2s. 6d.

**MENTAL NURSING.** A Text-book specially designed for the instruction of Attendants on the Insane. By WILLIAM HARDING, M.D., M.R.C.P.Lond

The want of a complete book for the instruction of Asylum Attendants has been long felt, and it is with confidence that the publishers recommend this work to the managers of all Institutions for the Insane.

"Nothing could be better devised to serve as a text-book. The lectures are so simple and so instructive that they cannot fail to impress and interest readers. . . . Of great value to all nurses. . . . No institution should fail to supply their officials with copies of the work."—*Local Government Journal.*

Second Edition, with Addenda, Crown 8vo., Cloth, 2s.

**THE MOTHER'S HELP AND GUIDE TO THE DOMESTIC MANAGEMENT OF CHILDREN.** By P. MURRAY BRAIDWOOD, M.D., formerly Senior Medical Officer to the Wirral Hospital for Sick Children.

"The book is written plainly and without pedantry, and the directions given for the management of the child are sensible and discreet. . . . We do not doubt the need of it in the ignorance of many mothers, and we can recommend it as likely to enlighten that ignorance."—*Glasgow Medical Journal.*

### "THE BURDETT SERIES" OF POPULAR TEXT-BOOKS ON NURSING

Ready early in February. Pocket Size, Price 1s. each.

No. 1.

**PRACTICAL HINTS ON DISTRICT NURSING.** By Amy Hughes, Superintendent of Nurses, Bolton Union Workhouse; late Superintendent of the Central Training Home, Queen Victoria's Jubilee Institute for Nurses for the Sick Poor.

No. 2.

**THE MATRON'S COURSE.** An introduction to Hospital and Private Nursing. By Miss S. E. ORME, Lady Superintendent, London Temperance Hospital.

London: THE SCIENTIFIC PRESS (Ltd.), 28 & 29, SOUTHAMPTON STREET, STRAND, W.C.

